# From Reward to Anhedonia-Dopamine Function in the Global Mental Health Context

**DOI:** 10.3390/biomedicines11092469

**Published:** 2023-09-06

**Authors:** Birgitta Dresp-Langley

**Affiliations:** Centre National de la Recherche Scientifique, UMR 7357 ICube CNRS, Université de Strasbourg Hôpitaux Universitaires Faculté de Médecine, Pavillon Clovis Vincent, 4 Rue Kirschleger, CEDEX, 67085 Strasbourg, France; birgitta.dresp@unistra.fr or birgitta.dresp@cnrs.fr

**Keywords:** reward, dopamine, brain, addiction, stress, anhedonia, compulsive behavior, COVID-19 pandemic, mental health

## Abstract

When “hijacked” by compulsive behaviors that affect the reward and stress centers of the brain, functional changes in the dopamine circuitry occur as the consequence of pathological brain adaptation. As a brain correlate of mental health, dopamine has a central functional role in behavioral regulation from healthy reward-seeking to pathological adaptation to stress in response to adversity. This narrative review offers a spotlight view of the transition from healthy reward function, under the control of dopamine, to the progressive deregulation of this function in interactions with other brain centers and circuits, producing what may be called an anti-reward brain state. How such deregulation is linked to specific health-relevant behaviors is then explained and linked to pandemic-related adversities and the stresses they engendered. The long lockdown periods where people in social isolation had to rely on drink, food, and digital rewards via the internet may be seen as the major triggers of changes in motivation and reward-seeking behavior worldwide. The pathological adaptation of dopamine-mediated reward circuitry in the brain is discussed. It is argued that, when pushed by fate and circumstance into a physiological brain state of anti-reward, human behavior changes and mental health is affected, depending on individual vulnerabilities. A unified conceptual account that places dopamine function at the centre of the current global mental health context is proposed.

## 1. Introduction

The neurotransmitter dopamine fulfills a critical function in regulating the responses of the mesolimbic system, also known as the reward system, in the neural circuits of the mammalian brain [1]. The reward system governs and regulates responses ranging from pleasure and cravings to disgust and anhedonia, triggered by chemicals and other stimuli and guiding a larger proportion of our behavior than we may be aware of or are ready to admit [2]. In ancient times, the function of the reward system made the difference between life and death, because it made us and other species deploy most of our or their attention and behavioral effort towards things that were important for the survival of the species, such as food, sleep, and sex. Our ancestors did not have a supermarket around the corner but had to hunt for food and often had to deploy a considerable amount of time and energy to find it. An individual who found sweet fruit in the environment, for example, had better consume it quickly, in as large of quantities as possible, before another did. Ripe fruits have the highest sugar content and provide higher amounts of instant energy compared with other foods. The human preference for sweet nutrients may, indeed, be due to the evolutionary advantage that craving and eating high-calorie foods has caused. Responding selectively to survival-relevant stimuli, in the course of evolution, has become hardwired into the brain’s reward system [3]. Food, sleep, physical contact, and sex are primary stimuli that reinforce the neural connections of the reward system, and a craving for these primary stimuli is inherent in humans [4], as well as most other mammals [5]. The reward system is composed of brain structures that mediate the physiological and cognitive aspects of a reward. This involves neurobiological processes driving the brain’s capapcity to associate stimuli such as substances or activities with a pleasant and positive outcome. Reward-associated mechanisms explain why individuals search for initially positive stimuli again and again and why this can ultimately lead to compulsive behaviors and addicions that pose a threat to mental health. This narrative review discusses how functional changes in the dopamine circuitry hijacked by compulsivebehaviors affect the brain’s reward system and may ultimately lead to an anti-reward state in the brain that has multiple metabolic and behavioralconsequences. Pandemic-related adversities and the stresses they engendered [6], with the long lockdown periods where people in social isolation had to rely on food for comfort or digital tools to get feedback rewards via the internet, can be seen as major triggers of changes in motivation and compulsive reward-seeking behaviors worldwide. These are linked here to the pathological adaptation of dopamine-mediated reward circuitry, triggered and consolidated through functional links with brain structures controlling the circadian rythm and/or the responses to chronic stress. This offersplausible insight into why environmental adversity pushed individuals of all nations, during and after the pandemic, into compulsive behaviors that couldlead to sleep disorders, anhedonia (from the Greek language: “inability to feel or experience pleasure”), depression, and in extreme cases, suicidal ideation. This narrative review is based on references searched on PubMed using the keywords given here above or combinations thereof. The search led to a selection of 152 articles/books, including the most relevant, with 70 of the references pointing to the most recent insights published between 2020 and 2023. The keywords and corresponding number of titles are summarized in Table 1. A unified conceptual account that places dopamine function at the heart of the current global mental health context is givenprior to the conclusions.

## 2. The Brain’s Reward System

A reward requires the coordinated release of heterogenous neurotransmitters, where dopamine plays the central role by mediating the reward value of food, drink, sex, social interaction, or specific substances or stimuli, as in abuse and addiction [1,7]. Moreover, the dopaminergic reward pathways of the brain influence the circadian rhythm [1], which is translated by the fact that reward-related activities such as feeding, exercise, sex, substance use, and social interactions produce elevated level of dopamine and alter the circadian rhythm of the central nervous system [1,8]. This explains, for example, why individuals on a “dopamine high” feel that they need less sleep [9,10], which is deceiving and, ultimately, has negativeconsequences on the brain and behavior [11]. In addition, the insidious process that leads from the initial subjective pleasure associated with a stimulus to the pathological craving that is characteristic of addiction is, ultimately, followed by a negative reward response. This leads to the progressive consolidation of an anti-reward state in the brain, as will be explained further below.

### 2.1. Reward

A reward is what we are all after every day, since the dawn of time, governed by a natural process where the brain associates so-called stimuli (chemical or cognitive) with a positive, desirable, outcome [12,13,14]. As a consequence, individual cognition and behavior adjusts to these outcomes by forming expectations and by searching for stimulations based on such expectations. The discovery of the brain’s reward system harks back to experiments from 1954 [15]. Scientists implanted electrodes in the brains of rats in order to stimulate specific areas of the brain with light electrical currents. The electrodes were activated by a lever that the rats could pull themselves. The researchers were able to observe how the rats pressed the lever up to 2000 times within an hour to receive the internal electric stimulation and for several hours until they were completely exhausted. Primary reinforcements such as food and sleep became less attractive than the stimulation that followed the lever pressure. Animal studies have also shown that there are reward circuits in the brain that originate in the evolutionarily ancient midbrain and connect with other brain areas [12]. One of the most important messengers in these regulatory circuits is the neurotransmitter dopamine. The major dopaminergic pathway involved in a reward is the so-called mesolimbic system [1], with midbrain dopamine neurons of the ventral tegmental area (VTA) projecting to the striatum, prefrontal cortex, amygdala, hippocampus, andother structures of the limbic system [1,3,16,17]. When rewarding stimuli are experienced, the dopaminergic mesolimbic system responds and initiates the release of dopamine. Dopamine is primarily released when a reward comes as a surprise or when stimuli appear that indicate a reward [17,18]; it is not, as such, responsible for the positive feelings during consumption of the reward. The positive feelings are essentially mediated by serotonin and endorphins [19] through interactions with the endocannabinoid system, which influences dopaminergic and serotonergic neurotransmission by generatingneuromodulatory effects at both the cellular and circuit levels [19]. Technological advances that facilitate the precise identification and control of genetically targeted neuronal populations may soon reveal more about the complex functional links between these systems and their potential relevance for motivated behavior and a reward response. There is no doubt that dopaminergic and opiodergic reward pathways are critical for survival. They provide the brain’s drive for eating, love, and reproduction, the so-called “natural rewards”. While this involves the release of dopamine in the nucleus accumbens and frontal lobes, dopamine is not the only reward transmitter. Dopamine antagonists and lesions of the dopamine system do not inevitably affect reward responses inthe nucleus accumbens or frontal cortex or the rewarding effects of apomorphine [17]. The brain’s reward circuitry ismultisynaptic; dopamine is the critical link in this circuitry [1,13,17].

### 2.2. Craving and Addiction

The release of dopamine can likewise be produced by “unnatural rewards” such as alcohol, cocaine, methamphetamine, heroin, nicotine, marijuana, and other substances or by compulsive activities such as gambling, eating, sex, and risk-taking behaviors [18]. Drugs can trigger a particularly powerful release of dopamine compared to the primary reinforcers or “natural rewards”. While the primary reinforcers may increase the dopamine levels by as much as 100%, drugs like cocaine can spike the dopamine levels by as much as ten times that amount. Strong increases in dopamine release are associated with particularly high reward sensations [18,20], i.e., there appears to be a direct correspondence between dopamine increase and the intensity of a pleasurable experience. However, a saturation effect sets in rather quickly in the case of primary reinforcers and no more dopamine is released, which is not the case with chemical drugs in the initial phase of the development of dependence [17,18,20]. Chemical drugs interfere with the reward system by tapping the dopamine circuitry more directly, and the typical course of an addiction development is characterized by resorting to the addictive substance more and more frequentlyas a consequence [20]. Likeinexcessive sugar or alcohol consumption [21], compulsive reward-seeking ultimately leads to cravings, a core characteristic of eating disorders and all forms of addiction [22]. Cravingsare associated with changes in the activity of the brain’s reward system, which not only preferentially responds to the target but also to the context directly associated with the habit. This can be specific places, moments in time, or social cues and people (consuming with friends). As the individual’s attention becomes increasingly focused on the drug, other primary reinforcers lose their appeal. The person is then interested in little else but the drug craved; in other words, the reward system is “hijacked” by the drug [20,21]. Impaired control over the compulsive drug-taking behaviors that characterize addiction may be due, in part, to specific dysfunctions in the frontal regions of the brain in drug abusers, where lower striatal dopamine (D2R) significantly correlates with lower brain glucose metabolism in the prefrontal cortex, such as the orbifrontal cortex [21]. The latter is involved in stimulus salience, and its deregulationhas been functionally linked to compulsive behavior [23].

### 2.3. Stress and Anhedonia

As an addictive behavior becomes more and more of a habit that cannot be controlled by willpower, the reward system becomes increasingly networked with other brain areas that control different aspects of habitual behavior [20,24]. This connectivity may explain why addicts relapse without any conscious decision to use again, as the addictive behavior is triggered more or less automatically by certain stimuli beyond individual conscious awareness [25,26,27]. Brain dopamine not only plays a critical role in the subjective pleasures associated with positive “natural” rewards but also in the objective reinforcement of behaviors and motivations associated with food, alcohol, and other drug rewards in addiction [27,28,29,30,31,32], involving synaptic plasticity in the brain circuitries that govern learning and memory. A recent review of the pertaining literature has found that anhedonia plays an essential role in the pathogenesis of both addictive and mood disorders and possibly in their cooccurrence within a single individual [33,34]. This can be explained on the basis of interactions between the reward system and other brain areas in terms of complex and still not very well-known processes that lead from the initial subjective pleasure associated with a drug stimulus to the pathological cravings characteristic of addiction, ultimatelyreversing into a negative reward response in the brain through the mechanisms of pathological adaptation. The underlying anti-rewardcircuitsseem to involve intertwined neuronal signaling in the medial prefrontal cortex and the lateral habenula, established during the course of development of addiction thatproduce aversive, stress-like states [35]. The progressive consolidation ofsuchanti-reward circuitry ultimately produces astress-related psychological state called *anhedonia* [36,37]. The “dopamine hypothesis” originally thought to be simple is now recognized as involving such complex circuit interactions, governing attention, reward expectancy, motivation, and ultimately pathological neuroadaptation [38], where excessive use is caused by decreased dopamine levels and the blunted responsivenessof the brain’s reward system. Moreover, dopamine has been intimately linked to the brain genesis of *anhedonia* through its interaction with the glucocorticoid system, responding to chronic stress. Prolonged stress leads to inflammatory processes that negatively impact functional connectivity in the corticostriatal (dopaminergic) reward circuits and symptoms in close interaction with the endocannabinoid and orexin systems [37], thereby affecting the synthesis and release of dopamine [37,38]. Through this process, the stimuli and activities we once enjoyed doing and that gave us pleasure become aversive and, ultimately, detrimental to our well-being. *Anhedonia*, i.e., the inability to experience pleasure, is thus a direct consequence of our brain’s response to chronic stress [37,38,39,40] and a main hallmark of chronic stress-induced depression [39]. Preclinical studies on rats [41] have shown that acute or intermittent stress increases the binge-like self-administration of drug rewards and dopamine response in the nucleusaccumbens, while prolonged stress decreases exploratory behavior and suppresses dopamine akin to the deterioration of the reward processes in anhedonia. It has been claimed further that, by leading to changes in the corticotropin-releasing factor in the brain, stress inevitably impacts all three stages of the addiction cycle (binge/intoxication, withdrawal/negative affect, and preoccupation/anticipation), exposing the individual to an emotional allostatic load and generating an allostatic state [42]. Allostasis would thus be the trigger forgrowing reward system pathology under the influence of dysregulated neurocircuitry for basic motivation. When alterations in its social environment challenge the biological balance of ananimal interfering with its homeostasis, stress will develop. This process is accompanied by changes in the peripheral cytokines released from various cell types, such as macrophages, T cells, and fibroblasts, highlighting the role of the innate immune system in the development of stress-induced psychiatric disorders like depression or depression-like behavior in animals [43,44]. Inflammatory responses and their effects on the brain are receiving increasing attention for their potential role in anhedonia and depression. The exogenous administration of inflammatory stimuli to humans and laboratory animals affectsthe neurotransmitters and neurocircuits involved in reward processing, including the ventral striatum and ventromedial prefrontal cortex, frequently associated with reduced motivation [45,46]. Increased inflammation (elevated inflammatory cytokines) has been reported in a significant proportion of patients with depression and other psychiatric conditions involving anhedonia [46].

## 3. Behavioural Implications

The important role of dopamine in the neurocircuitry of the reward system and its interactions withotherbrain systems provide a functional (brain-based) interpretation of the changes in health-relatedbehavior in light ofthe conceptual framework of the neurobiology of motivation. The resources of modern neuroscience offer an opportunity to conceptualize the dopaminergic neurocircuitry and its multiple interactions with other pathways, functions, and mechanisms in healthy rewards; in addictive processes; and in the development of chronic stress, anhedonia, and ultimately, depression. Changes in motivation that are associated can be generalizedforother psychopathological processes. Therein, compulsory reward-seeking and, ultimately, addiction may be conceptualized as a cycle of decreased function of the brain’s reward system and the recruitment of an anti-reward system. As a consequence, the normally homeostatic function of reward fails, as compulsivereward-seekingin the shortterm results in the amelioration of a sudden reward deficit but, ultimately, in the long term, produces neurochemical dysregulations ofdecreased dopamine and opioid peptide function, increased corticotropin-releasing factor, and a chronic deviation of the reward system that is fueled not only by dysregulation of the reward circuits but also by the recruitment of other neurotransmitter and hormonal stress responses [46,47]. Once this is understood, we have the keys fora functional explanation of the behavioral changes in individuals and groups observed under conditions of adversityorthe influence of environmental stressors.

### 3.1. Compulsive Behavior

Compulsive behavior consists of repetitive acts driven by the urge that one has to perform them while aware that they are not in line with one’s overall goal [48]. There is an initial relationship between vulnerability to impulsivity or a lack of impulse control in terms of self-regulation [49] and compulsive behaviors as a consequence of impulsivity in both health and addiction [22,50]. The neural systems basis of compulsive behavior involves dopaminergic, as well as serotoninergic, mechanisms within the corticostriatal circuitry [51,52,53]. Also, increasing evidence supports a role of the noradrenergic system in compulsive behavior [54,55], with an involvement of noradrenergic mechanisms in the regulation of the glucocorticoid levels [56,57], which have been linked to stress-induced repetitive behaviors [58]. A proven role of noradrenergic interactions in anxiety, coping, and impulse control [59,60,61,62] contributes to both the emergence and increase in severity of compulsions [63,64,65]. To attempt to clarify the concept of vulnerability to impulsivity, individual or population vulnerability is to be defined as an outcome of other concurrent variables in a potentially preventable, reversible process [66]. A vulnerable population may consist of individuals with activity-limiting physical or mental impairments or of socially or economically disadvantaged people such as homeless, rural, adopted, or the elderly. It may consist of racial/ethnic minorities, gender minorities, religious minorities, children with parents who are activeduty members of the armed forces, veterans, or others. Vulnerability needs to be considered in terms of its severity in light of specific characteristics, circumstances, or combinations thereof. The concept of vulnerability is important in public health. Vulnerable individuals often resort to alternative means of coping with stressors compared with more resilient individuals and may therefore be at a higher risk of developing extreme reward-seeking behaviors in the form of substance or behavioral addictions. This relationship can be explained by the neuroplasticity of the circuits involved, where the plasticity-dependent deregulation of dopamine interactions in the brain accounts for how circuits may gradually become dysregulated, stay dysregulated, and then promote further vulnerability to further the dysregulation [67].

### 3.2. Craving and Bingeing

A craving is a particual aspect of compulsive behaviour, andbingeing is a direct result of craving. Preoccupation, a motivation-related mental state triggered by aversive internal or external stimuli, may produce compulsive reward-seeking in healthy and in addictive behaviours [68,69,70] that ultimately results in cravings and bingeing. Human neuroimaging studies attribute a key role of the prefrontal cortex (orbitofrontal, medial prefrontal, and prelimbic/cingulate) and the basolateral amygdala tocue-induced cravings and bingeing as the result. A primary disruption ofthe frontal brain regions ordisruptive effects secondary to changes in the striatal dopamine activity may affect dopamine cell activity. Different types of dopamine neurons connect with distinct brain networks, ensuring distinct roles in motivation regulation and control. Some of themdirectly supportbrain networks for seeking, evaluation, and value coding and memory. Others encode motivation-relevant salience, supporting the brain networks for general motivation and cognitive integration [70]. In extreme reward-seeking behavior, an increase in severity of the compulsion leads to craving and bingeing, which are often, but not always, relating to a specific stage of the addiction cycle [70,71], where neuroplasticity intervenes with a change in the firing activities of mesolimbic dopamine neurons during the initial stimulus (substance or other drug) exposure and is then translated to engagement of the dorsal striatum, disruption of the frontal system function, and recruitment of the brain’s stress systems [72]. Similar to what happens in the case of drug abuse, chronic, repeated stimulation of the dopaminergic system in NAc by palatable food and their associated cues shifts signaling to the dorsostriatal dopaminergic pathways, which results in habit formation for compulsive eating and reflects a maladaptive, stimulus-driven habit that overrides voluntary goal-directed behavior [27,72]. Through the process of consolidation, this produces a powerful drive for food- or drug-seeking behavior even months after dieting or drug withdrawal and provides an explanation in the brain for relapse due toaddiction. Relapse following withdrawal is the result of healthy neuroadaptive processes gone wrong [28], or pathological adaptation, in the central nervous system, opposing the acute reinforcing actions of the drug via a deregulation of the mechanisms that mediate positive reinforcement. Such deregulation explains the emergence of anxiety, anhedonia, and depression during withdrawal.

### 3.3. Negative Affect

Motivational changes related to the sudden withdrawal of a reward [58,59] in both those healthy and addicted produce negative affects and changes in mood. These can lead to anhedonia [58], depression, and in the extreme, suicide. A negative affect is closely linked to the brain’s stress systems [73] involving the corticotropin-releasing factor (CRF), which interacts with the dopaminergic brain circuitry. The primary physiological stress response is partly mediated by the hypothalamic–pituitary–adrenocortical (HPA) axis in mammals, with stressors activating the central nervous system and the hypothalamus and therefore the CRF inthe pituitary gland. The pituitary gland will, in turn, release adrenocorticotropic hormone (ACTH), which induces glucocorticoid synthesis and release from the adrenal tissue into the blood. The glucocorticoids will then affect the target tissues throughout the body, as each step of the HPA axis is self-regulated by an array of feedback loops. This explains why numerous other neurotransmitter systems, such as thedynorphin, NPY, substance P, nociceptin, and orexin systems, interact with the brain’s stress systems. Stressful situations are an unavoidable element in everyday life. Stressors activate a number of complex mental and physiological reactions in anorganism [74], thus affecting the state of health of an individual. Stress is the main risk factor in the development of sleep disorders, depression, addiction, and other health issues that result from the addiction [75,76]. Reward-related behavior in stressed individuals affects the dopamine levels and dopaminergic neuronal activity in the mesolimbic system. Changes in mesolimbic dopaminergic neurotransmission regulate coping with stress through the adaptation of behavior to environmental stressors in their context. Modulation of the dopaminergic reward system presumably drives the selection ofthe optimal coping strategies. Aversive events may negatively regulate the dopaminergic reward system and perturbthe reward sensitivity; the latter is known to be associated with chronic stress related depression [76,77]. The mesolimbic dopamine system is not only activated by reward but also by aversive stimuli (stressors), adding further complexity to the functional links between stress, the reward response, and the reward system [77]. Internal factors altered by stress may produce drug addiction vulnerability [78] by the neuroinflammatory, neurotrophic, and neurotransmitter factors impacting the mechanisms of craving and relapse susceptibility and linked to sleep disorders [74,78] and depression [79].

### 3.4. Sleep

While healthy, positive stimulation of the reward system promotes healthy sleep, compulsive reward-seeking, on the other hand, leads to the asynchronization of the circadian rhythm [80,81,82,83], centrally controlled by serotonin and responsible for dysfunctional sleep patterns. Pathological changes in sleep patterns and behavior are a product of pathological brain adaptation involving antagonistic processes (*allostasis*) that lead to irritability, *dysphoria*, anxiety, and *anhedonia* in what is sometimes called the brain’s anti-reward system [80,81,82,83,84,85,86]. Because compulsive reward-seeking literally usurps everyday behavior, i.e., eating, sexuality, exercise, and others, it “hijacks” the natural effects of substances or activities that produce pleasure through the release of dopamine [1,12,17]. A natural reward at first decreases the reward threshold, while the chronic addiction to a substance or a behavior increases this threshold, hence the need to consume more of a given drug to reach it. Compulsive behavior leads to addiction by increasing the concentration of extracellular dopamine. This process involves a complex chain of specific mechanisms. These have been reported to include a decrease in the inhibitory tone exerted by GABAergic neurons on dopaminergic neurons, the release of opioids and endogenous cannabinoids, as well as direct effects on dopaminergic neurons resulting in an increase in the neural firing frequency [31,32]. Dopamine not only mediates pleasurable effects. It is also involved in complex phenomena relating to the attribution of an “added value” or “incentive value” to a substance, situation, or behavior. Contextual cues associated with a drug then replace the value of the drug itself as the result of a conditioning process that precipitatesa relapse. Contextual cues then produce the overwhelming, urgent, and irrepressible desire to resort to the drug againand again [13,14,17,31]. This process is related to changes in the amygdala [32,33] resulting in negative emotional states and sleep disruption [86,87]. Most importantly, sleep deficiency promotes further vulnerability to compulsive behaviors and addictions, which is now recognized as a worldwide problem [88].

## 4. COVID-19 Pandemic and Mental Health

In 2022, the World Health Organisation (WHO) issued a brief [89] with facts and figures showing that the COVID-19 pandemic has had a severe impact on the mental health and well-being of people around the world. While many individuals have adapted, others have experienced mental health problems that, only in some cases, are a consequence of COVID-19 infection. The brief stated further that the pandemic also continues to impede access to mental health services and raised concerns about increases in suicidal behavior, stressing the causal role of psychological and environmental factors producing ontological insecurity in individuals and populations. In the wake of the COVID-19 pandemic, experts havewitnessed a

➢ significant rise in addictions and related mental illnesses;
➢ significant rise in the corresponding anti-depressant prescription uptake;
➢ increased risk of suicidal ideation or suicide.

Any potential doubts about the impact of COVID-19 on mental health have been largely put to rest by the most recent papers on the topic [90,91,92,93,94]. Loneliness, physical exhaustion, sleep disturbances, and *anhedonia* linked to compulsive or addictive behaviors such as binge-eating [95,96], substance abuse [97], and digital reward craving [98] were identified asbeing linked to suicidal ideation [95,99,100]—in particular, in younger individuals—during and after the pandemic. These reflect some of the most negative effects of pandemic-related adversity on mental health worldwide.

### 4.1. Adversity and Vulnerability

Mental health issues arise from a specific context. Recently reported effects of the pandemic have shown, beyond all reasonable doubt, that adversity affects people’s capacity tocope with the stresses it produces. Conditions of extreme adversity challenge the stress and immune system responses needed for coping, and beyond a certain threshold, such conditions can trigger behavioral changes as listed and discussed here above; all of them have been functionally linked to the complex brain pathways involving dopamine release, reward mechanisms, and their interactions or regulations [77,79]. During the different waves of the pandemic, many individuals had an actual COVID-19 infection, with direct effects on the brain’s immune system, stress centers, and the dopamine system, affecting people’s mood beyond the actual duration of the illness. Cohort studies examining the psychopathological and cognitive status of COVID-19 pneumonia survivors [101], for example, have shown that three months after discharge from the hospital, close to 40% still self-rated their symptoms in the clinical range for at least one psychopathological dimension, with persistent depressive symptomatology, while anxiety and insomnia progressively decreased during follow-up. Studies on the impact of the COVID-19 pandemic on individuals with eating disorders have been performed all over the world, showing that the traumatic effects of COVID-19 have exacerbated specific eating disorder-related psychopathologies [102], pointing towards complex interactions between environmental and personal factors. Healthy siblings of patients with eating disorders have beenfound to present specific psychological vulnerabilities, i.e., specific associations between interpersonal sensitivity and posttraumatic symptoms, including anxiety, depression, and compulsive behaviors [102,103]. Moreover, having to cope with adversity during childhood increases the risk of mental health problems in adulthood, and it has been suggested that this effect may be mediated by increased striatal dopamine neurotransmission [104]. Also, while resilience may be viewed as an individual’s positive adaptation to experiences of adversity [105], it is also known that conditions of adversity reinforce the already exisiting vulnerability to mental illness and may create a new vulnerabilty duringspecific adverse contexts such as a pandemic [92] or armed conflict [106]. It has been confirmed that stress responses can cause functional changes in the amygdala and the dopaminergic circuits of the brain in relation to stress-induced cortisol release mechanisms [106,107,108]. Some of these studies have shown a cortisol-induced increase in serotonin in subjects with major depressive disorder, offering further insight into the functional links between stress, sleep disorders, and depression, where an increase in one brain correlate affects another, thereby creating or increasing the physiological vulnerability to mental illness in individuals under prolonged stress.

### 4.2. Bingeing

The COVID-19 pandemic creatednegative health consequences such as consuming high-fat and sugar foods, increasing body weight [109], anxiety and depression [110], anhedonia [110,111], or a combination thereof [83]. As a core characteristic of eating disorders and all forms of addiction [22], bingeing is associated with changes in the activity of the brain’s reward system in the process of pathological adaptation of the central nervous system through deregulation of the reward mechanisms generating positive reinforcement. As explained earlier, this deregulation progressively leads to the consolidation of the anti-reward brain state that produces anxiety, anhedonia, and depression. Most people during COVID-19 lockdown reportedly turned to substances or other “rewarding” activities to deal with the stress of isolation and the negative feelings it engendered [112,113]. A number of factors related to the COVID-19 pandemic may have contributed to the reportedly high incidence of food addictions during and after that period [113,114]. It has been suggested that people under the influence of the excessive and uncontrolled consumption of extremely palatable foods experience similar highreward sensations as recorded for addicts resorting to classic intoxicants, both at the behavioral and neurobiological levels [88,113,114]. As lives and daily routines were transformed by the pandemic and rapidly rising anxiety fueled by social isolation and, often, unemployment, the mental health of millions is at stake. In the United States alone [115], close to 60% of adults screened declared that the pandemic led them to abuse alcohol and other drugs, binge eating, and/or compulsive internet gambling.

### 4.3. The New Digital Drug

**Figure 1:**
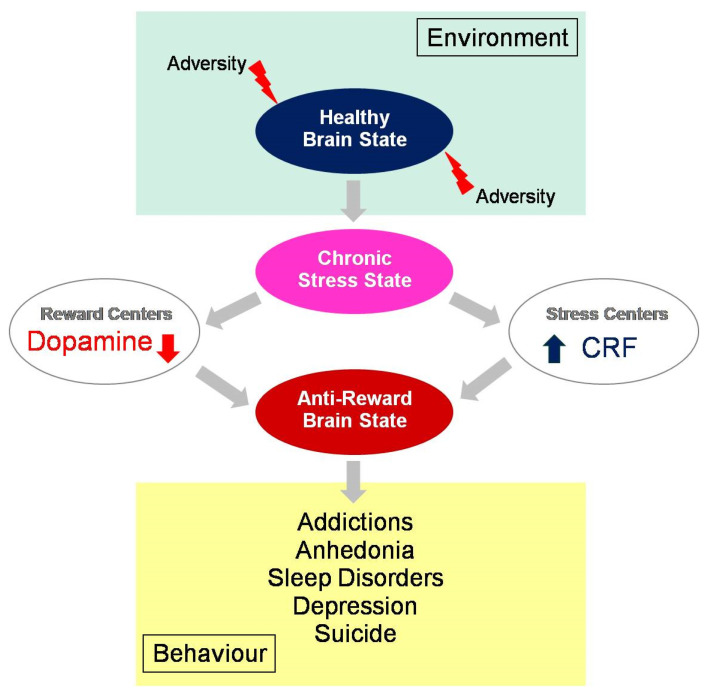
Behavioral consequences of an anti-reward brain state, with ultimately depleted dopamine levels in the reward centersand increased cortisol levels in the centers that regulate the brain’s response in pathological adaptation to chronic stress. Under adverse environmental conditions, such as those generated by the COVID-19 pandemic, healthy reward-seeking has progressively evolved into stress-related compulsory behaviors and/or addictions leading to anhedonia and related mental health issues worldwide.

COVID-19 spread across the world ata rapid pace, and to further limit the spread of infection, lockdowns were declared in most parts of the world. People were forced to stay indoors, and the internet was the only source of entertainment. This context engendered addictive behavior with measurable negative effects on anxiety and sleep quality, especially among younger individuals [115,116,117,118]. Digital addiction involves theinternet as a channel through which individuals may access whatever content they want (games, social media, shopping, etc.) wherever and whenever they want. The development of the addictive response is digitally facilitated by such instant availability [117,118,119,120,121,122]. At an advanced stage, digital addiction [118] is associated with significant and permanent symptomatic psychological, cognitive, and physiological states, with a measurable dopamine deficiency and impaired mental health [121]. Psychological stress [121,122,123,124], anxiety and depression [125,126,127], eating disorders [128,129,130], sleeplessness [131,132], and mood changes, along with suicidal ideation [133,134], are the most frequently reported. A compilation of cross-national studies on more than 89,000 participants from 31 nations performed almost ten years ago, well before the COVID-19 pandemic, already suggested a global prevalence estimate for digital addiction of 6% worldwide [120]. Interdependent variables such as sociocultural factors, biological vulnerabilities (genetic predisposition and preexisting metabolic disorders), and psychological factors (personality characteristics and a negative affect) play a critical role [135,136,137,138,139,140]. Excessive seeking of the new digital drug and addiction to the internet have been identified as part of the consequences of the COVID-19 pandemic, with a major impact on the mental health of predominantly young people and teenagers [138,139,140,141,142,143,144,145,146]. This involves, like all compulsive behavior loops, the brain’s reward circuitry, with all the complex interactions between environmental, metabolic, and neurobiological changes in the brain discussed above. Dopamine, and the modulation thereof under conditions of adversity and stress (Figure 1), is the common functional denominator [147].

## 5. Conclusions

In her book *Dopamine Nation* [148], the psychiatrist Anna Lembkeexplained why the relentless pursuit of pleasure leads to addictions and, ultimately, unhappiness and pain. In this paper, the new information on dopamine-regulated rewards in the genesis of compulsive behaviors, chronic stress states, and mood changes is linked to the most recently reported mental health consequences of the COVID-19 pandemic. Dopamine regulates healthy reward-related behaviors by producing a “dopamine high” in the mesolimbic dopaminergic pathways of the brain. The transition from healthy reward-seeking to compulsive behaviors and chronic addition, however, is linked to a deregulation of the brain’s reward curicuitry in interactions with other brain systems involved in responding to stress. Chronic stress, in return, affects the dopamine levels and dopaminergic neuronal activity, ultimately generating a “dopamine low” or anti-reward brain state linked to anhedonia and depression. By regulating the behavioral responses to environmental stimuli, dopaminergic neuromodulation, ultimately, is a unifying brain explanation for healthy and pathological processes of coping with stress. Adversity, as a consequence of the prolonged stressful events engendered by the COVID-19 pandemic and correlated with increasingly compulsive reward-seeking worldwide, has perturbed our overall reward sensitivity and produced chronic stress-induced depression and other mental health problems across populations and nations. As a central neurotransmitter at the heart of an intriguingly complex relationship between pain and pleasure, i.e., stress and the brain’s reward system, it ensures a critical function in the slow process that leads from a healthy reward response to the brain state of anti-reward, currently a major global mental health challenge worldwide.

Future research on dopamine interactions, from the molecular, cellular, and integrative brain circuitry levels, and their underlying behaviors has acquired a novel global significance in this new mental health context [89,143,146]. This paper prompts further investigations into the still underexplored links between dopamine-mediated reward functions, serotonin functions, inflammatory responses, hormonal functions, and their implications for pathological brain adaptation.

## Figures and Tables

**Table 1:** Number of selected titles (references) based on the keyword search in PubMed.

Keyword	Titles (*n*)
Reward	27
Dopamine	21
Brain	21
Addiction	47
Stress	23
COVID-19	19
Anhedonia	10
Behavior	16
Compulsive	12
Mental Health	11

## Data Availability

All relevant data can be found in the literature cited in this communication.
